# Bioelectrocatalytic Activity of W-Formate Dehydrogenase Covalently Immobilized on Functionalized Gold and Graphite Electrodes

**DOI:** 10.1021/acsami.0c21932

**Published:** 2021-03-03

**Authors:** Julia Alvarez-Malmagro, Ana R. Oliveira, Cristina Gutiérrez-Sánchez, Beatriz Villajos, Inês A.C. Pereira, Marisela Vélez, Marcos Pita, Antonio L. De Lacey

**Affiliations:** † Instituto de Catálisis y Petroleoquímica, CSIC, c/Marie Curie 2, L10, 28049 Madrid, Spain; ‡ Instituto de Tecnologia Química e Biologica, Universidade Nova de Lisboa, Apartado 127, 2781-901 Oeiras, Portugal

**Keywords:** carbon dioxide reduction, formate dehydrogenase, bioelectrocatalysis, oriented immobilization, metalloenzymes

## Abstract

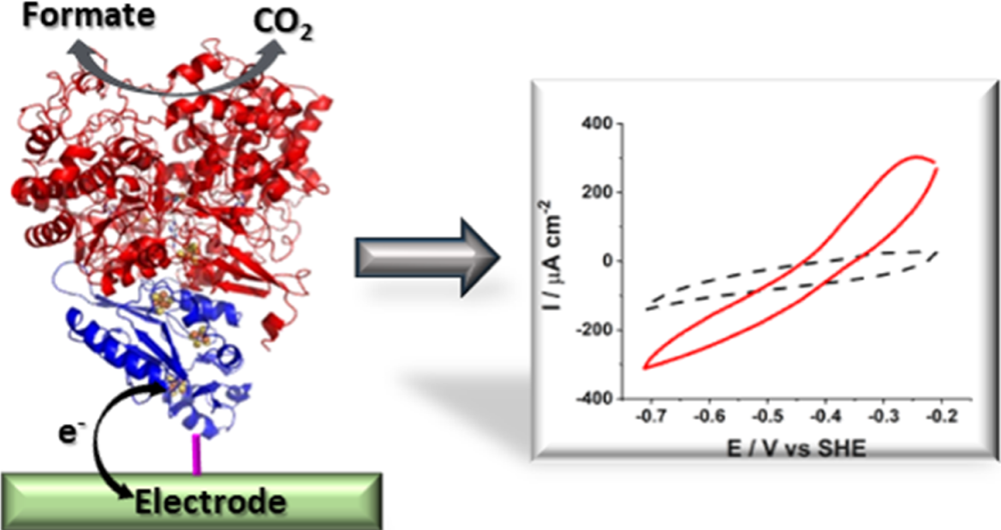

The decrease of greenhouse gases such as CO_2_ has become a key challenge for the human kind and the study of the electrocatalytic properties of CO_2_-reducing enzymes such as formate dehydrogenases is of importance for this goal. In this work, we study the covalent bonding of *Desulfovibrio vulgaris* Hildenborough FdhAB formate dehydrogenase to chemically modified gold and low-density graphite electrodes, using electrostatic interactions for favoring oriented immobilization of the enzyme. Electrochemical measurements show both bioelectrocatalytic oxidation of formate and reduction of CO_2_ by direct electron transfer (DET). Atomic force microscopy and quartz crystal microbalance characterization, as well as a comparison of direct and mediated electrocatalysis, suggest that a compact layer of formate dehydrogenase was anchored to the electrode surface with some crosslinked aggregates. Furthermore, the operational stability for CO_2_ electroreduction to formate by DET is shown with approximately 100% Faradaic yield.

## 1 Introduction

The increase of CO_2_ atmospheric concentration plays a major role in climate change and global warming. The greenhouse effects caused by current emissions may be irreversible for at least 1000 years after such emissions have stopped.^1^ A negative emission balance that reduces the amount of CO_2_ is one of the most important challenges ever faced by the human kind. CO_2_ has intrinsic kinetic inertia and high thermodynamic stability making it difficult to either trap or reduce. Formic acid/formate is a stable product that can be obtained by bi-electronic reduction of CO_2_ (eq 1). It has several advantages because it is liquid at standard temperature and is a good energy vector that can be stored easily and then employed in different energy processes such as in fuel cells or synthesis of chemicals.^2^
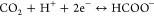
1

**Figure 1:**
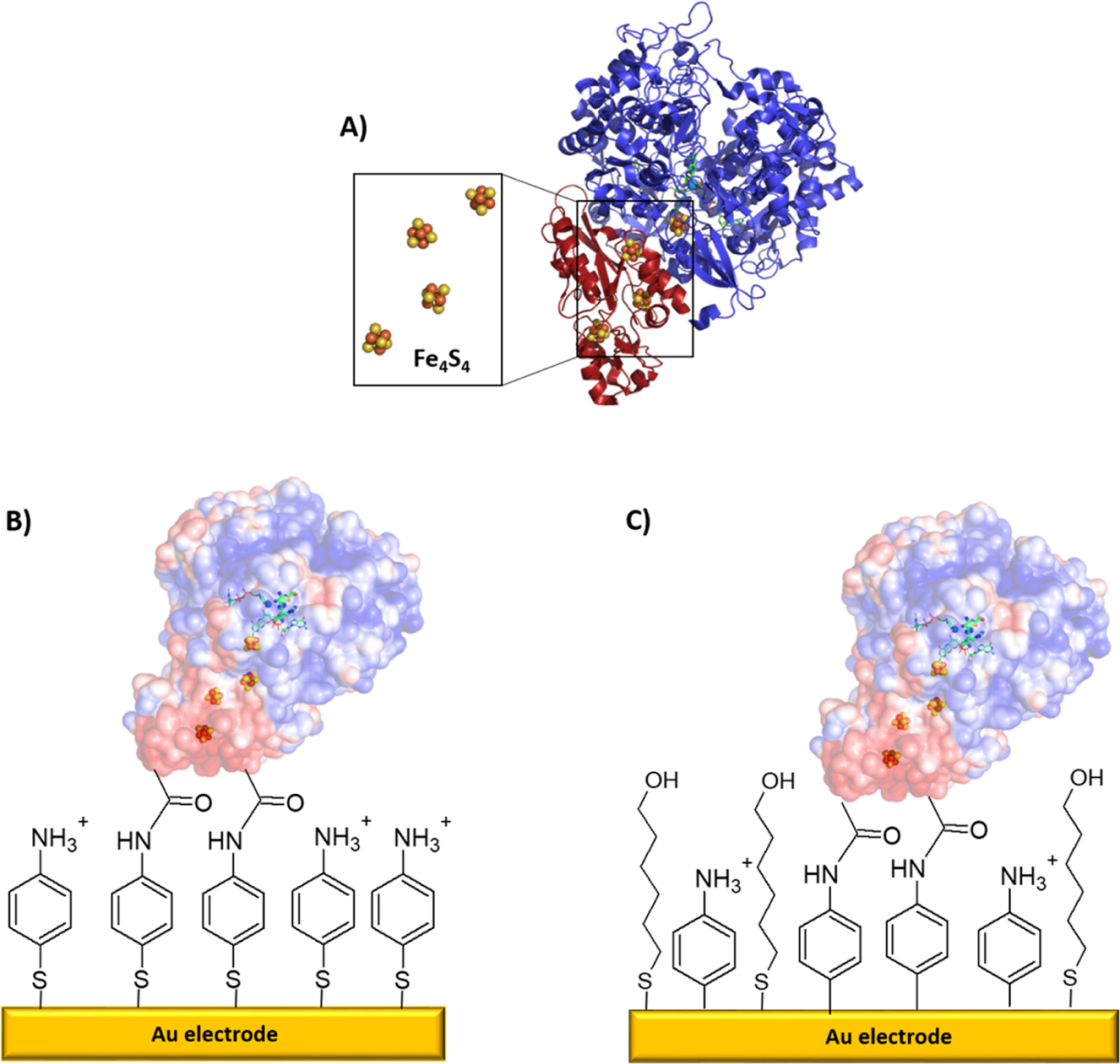
(A) Representation of the crystallographic structure of *Dv*H-FDH with its exposed Fe_4_S_4_ cluster. Covalent immobilization of *Dv*H-FDH on modified gold electrodes with a (B) 4-ATP SAM and (C) a mixed AP + MH layer. In (B) and (C) the electrostatic potential mapping of the protein surface is shown. Reprinted in part with permission from ref (13). Copyright 2020 American Chemical Society.

Electrochemical reduction of CO_2_ has been demonstrated, but it requires applying a very high overpotential that implies low selectivity, low catalytic yield, and a lack of specificity.^3^ Interestingly, formate dehydrogenases (FDH) are redox enzymes that specifically catalyze the reduction of CO_2_ to formate in a reversible way. Therefore, their study as bioelectrocatalysts is of importance as a source of inspiration for obtaining more efficient and sustainable biomimetic catalysts for CO_2_ reduction. There are two types of FDH enzymes: the first type is formed by NAD^+^-dependent FDHs, which are present in plants, fungi, and some aerobic bacteria; these FDHs are mostly biased toward formate oxidation.^4^ The second type comprises Mo- and W-dependent FDHs, which are present in anaerobic prokaryotes and have shown high efficiency for reducing CO_2_ to formate with high turnover values. In order to function *in vitro*, these FDHs require strict anaerobic conditions and an initial activation by reduction, which are the conditions for the enzyme to function *in vivo*.^5−7^ These kinds of FDHs are able to catalyze CO_2_ reduction with low redox overpotential, close to the thermodynamic value (*E*^0^′ = −0.42 V vs SHE). Specifically, W-FDH enzymes, due to the lower potential of their active site, seem to be the best candidates for catalyzing CO_2_ reduction.^8^ Several studies have been performed to elucidate the catalytic mechanism of FDHs.^9−12^ Recently, Oliveira et al. reported an efficient system to produce a recombinant W-containing FDH from *Desulfovibrio vulgaris* Hildenborough (*Dv*H-FDH), which is a heterodimer that consists of two subunits with molecular masses of 110.8 and 27.5 kDa, respectively. The active center of tungsten is located in the large subunit (Figure 1A) and is responsible for the reversible conversion between carbon dioxide and formate according to eq 1. The crystallographic structure model shows that the enzyme surface has a heterogeneous distribution of charges, in which the formate entrance site is positively charged, while the redox donor/acceptor binding site (containing a Fe_4_S_4_ cluster) is negatively charged. Another three Fe_4_S_4_ clusters within the protein structure form the electron-transfer path to the W-active site. Interestingly, the enzyme can be stably isolated under aerobic conditions and has a high catalytic turnover for a CO_2_ reduction of 315 ± 28 s^–1^ using excess of reduced methyl viologen as a reductant. It was also shown that 5 min pre-incubation of *Dv*H-FDH with 50 mM dithiothreitol was enough to observe full catalytic activity.^13^

These interesting biochemical properties of *Dv*H-FDH have spurred bioelectrocatalytic studies of CO_2_ reduction focusing on building interfaces for improving the rate of charge exchange between electrodes and the enzyme’s W active site. Miller et al. described the adsorption of *Dv*H-FDH onto metal oxides electrodes with fast direct electron transfer (DET) across the enzyme–metal interface.^14^ Szczesny et al. fabricated a gas diffusion electrode modified with polymer/enzyme layers for electroenzymatic CO_2_ reduction in which the low potential redox mediator was covalently bound to the polymer. This configuration avoided the disadvantages of using a redox mediator in solution.^15^

In the present work, we study different approaches to modify gold and low-density graphite electrodes in order to place amino groups on their exposed surface. These functionalities allow for the covalent anchoring of *Dv*H-FDH with an orientation modulated by electrostatic interactions to favor DET with the electrode while maintaining the operational stability. Both formate oxidation and CO_2_ reduction electrocatalytic properties of the immobilized *Dv*H-FDH have been studied, and atomic force microscopy (AFM) and quartz crystal microbalance (QCM) characterization allowed correlating the modified electrode conformation with their catalytic function.

## 2 Materials and Methods

### 2.1 Reagents

All used chemicals and materials were purchased from commercial suppliers and employed without any further purification. 4-Aminothiophenol (4-ATP) 97%, 6-mercapto-1-hexanol (MH) 97%, *p*-nitrophenyldiazonium tetrafluoroborate salt 97%, *N*-ethyl-*N*′-(3-dimethylaminopropyl)carbodiimide hydrochloride (EDC), *N*-hydroxysuccinimide (NHS) (97%), dl-dithiothreitol (DTT), glutaraldehyde (25%), sodium hydrogen carbonate, sodium formate >99%, methyl viologen dichloride hydrate (MV) 98%, benzyl viologen dichloride hydrate (BV) 97%, potassium cyanide (99%), potassium chloride ultrapure, ethanol absolute grade, phosphoric acid 99%, citric acid 99%, sodium citrate 99%, sodium hydroxide 99%, sulfuric acid >95%, 2-(N-morpholino)ethanesulfonic acid (MES 99.5%), tris(hydroxymethyl)aminomethane (Tris–HCl), sodium tetrahydroborate and tetrabutylammonium fluoride >99%, acetonitrile, and low-density graphite (LDG) (99.9%, 3 mm diameter) were obtained from Sigma-Aldrich (Merck). MicroPolish alumina suspensions was purchased from Buehler. Sodium dihydrogen phosphate 99%, hydrogen peroxide 30%, sodium hydrogen phosphate 99%, and Amicon Ultra-0.5 Centrifugal Filter Unit Ultracel-50 regenerated cellulose (RC) membrane, 0.5 mL sample volume were obtained from Merck. Sodium nitrate was obtained from Panreac and glycerol 10% from Scharlau.

All solutions were prepared with Milli-Q grade water (18.2 MΩ·cm, Millipore) and purged with 99.99% N_2_ (Air Liquide) for 10 min before any further use.

### 2.2 Enzyme

The recombinant tungsten-dependent FdhAB formate dehydrogenase from *D. vulgaris* Hildenborough (*Dv*H-FDH) was isolated and purified as previously described.^13^ 33.7 μM stock solutions, with a turnover of 1144 s^–1^ for formate oxidation with 2 mM BV and of 236 s^–1^ for CO_2_ reduction with 1 mM MV^+^· were stored at −80 °C in a buffer solution containing 20 mM Tris–HCl, 10% glycerol, and 10 mM NaNO_3_ at pH 7.6. Prior to a set of measurements, the buffer was exchanged to 10 mM MES at pH 6 by ultrafiltration with 50 kDa centrifugal filters, and the enzyme was stored in aliquots of 6 μL containing *Dv*H-FDH 27 μM at −80 °C.

### 2.3 Preparation of Gold and Graphite Electrodes

Disk electrodes of polycrystalline gold with 0.5 cm diameter (Pine Instruments) were cleaned by immersion in a freshly prepared *piranha* solution (3:1 H_2_SO_4_/H_2_O_2_) (caution: this is a very exothermic and hazardous solution) for 10 min and further rinsed with MilliQ water. Afterward, the electrodes were polished successively with alumina suspensions of 1, 0.3, and 0.05 μm in diameter, respectively, and sonicated for 10 min in a H_2_O/EtOH mixture (1:1) to remove alumina traces from the surface. The gold electrode surfaces were then electrochemically cleaned using two processes of cyclic voltammetry. In the first one, 15 cycles were performed between 0 and −1.5 V versus Ag/AgCl (3 M Cl^–^) at 0.2 V s^–1^ scan rate in a 0.5 M NaOH electrolyte solution. In the second step, 20 cycles between 0 and +1.5 V versus Ag/AgCl (3 M Cl^–^) at 0.2 V s^-1^ in 0.5 M H_2_SO_4_ were applied. The electroactive area of the electrodes was calculated, as described by Trasatti and Petrii,^16^ resulting in an average roughness of 1.1 ± 0.3.

LDG rods of 0.3 cm diameter were also used as working electrodes. One edge of each rod was polished for 60 s with an emery sandpaper, rinsed with MilliQ water, and then sonicated for 10 min in H_2_O. Finally, the clean electrodes were left to dry for 12 h at room temperature before use. The rods’ sides were covered with the Teflon band so that the geometric area of the LDG exposed to the solution was 0.07 cm^2^. The graphite rods were then fitted into a custom-built plastic adaptor screwed to a Pine Instruments rotating electrode system.

### 2.4 Chemical Modification of the Electrode Surface

Two different approaches have been used to modify the electrodes’ surface in order to expose positively amino groups for further covalent immobilization of *Dv*H-FDH. In the first approach, the clean gold electrode surface was modified with a thiol self-assembled monolayer (SAM) of 4-ATP as previously reported by us.^17^ In the second approach, the clean gold or LDG electrodes were electrochemically modified with 4-nitrophenyl radicals obtained by the electrochemical reduction of *p*-nitrophenyldiazonium tetrafluoroborate salt. This procedure was done as previously reported for LDG^18^ and gold^19^ electrodes. Only in the case of gold electrodes, the non-modified regions were blocked with self-assembled MH molecules.^20^

### 2.5 Covalent Immobilization of Formate Dehydrogenase on Electrode Surfaces

20 μL of 8.1 μM *Dv*H-FDH solution in 10 mM MES pH 6.0 buffer was placed on the modified electrodes’ surface and incubated for 30 min to allow for enzyme molecule rearrangement to yield an oriented binding through dipole moment-driven electrostatic interactions. Afterward, 20 μL of a 10 mM MES pH 6.0 buffer solution containing 7.7 mM EDC and 9.5 mM NHS was added on top for 90 min for covalent attachment of *Dv*H-FDH to the amino groups of the electrode surface. After that, the modified electrodes were rinsed by immersing them several times in 10 mM MES solution at pH 6.0. Finally, the immobilized *Dv*H-FDH was activated inside the glovebox by incubating the enzymatic electrodes for 5 min in a 10 mM MES solution at pH 6.0 containing 50 mM DTT. This procedure has demonstrated to increase the activity of the enzyme.^13^ Prior to any electrochemical measurements, the electrodes were washed again by immersing them several times in 10 mM MES solution at pH 6.0.

Additional glutaraldehyde cross-linking of FDH was also tested as follows: after activation with DTT, the modified electrodes were incubated for either 30 or 60 min in a 0.9% glutaraldehyde solution in 10 mM MES at pH 6.0. Finally, the electrodes were rinsed as described before.

### 2.6 Electrochemical Measurements

All electrochemical measurements were carried with an Autolab PGTAT30 potentiostat controlled by GPES 4.9 software (Eco Chemie, The Netherlands). All electrochemical experiments involving the FDH were performed inside an MBraun glovebox with an oxygen content lower than 0.1 ppm. Cyclic voltammetry and chronoamperometry were conducted in a three-electrode glass cell at 25 ± 1 °C thermostated with a circulating water jacket. Either polycrystalline gold disks or LDG rods were employed as working electrodes, while a saturated calomel electrode (SCE) and a flame-annealed platinum wire were used as a reference and counter electrodes, respectively. All potentials are given with respect to SHE (*E*_SHE_ = *E*_SCE_ + 0.241 V). The electrolyte volume in the experiments was 33 mL. Prior to any electrochemical measurements, any possible traces of oxygen in the solutions were removed inside the glovebox by bubbling N_2_ (99.999%) for 20 min and the flow of the inert gas was kept over the working solution during the measurements. All the given current densities were calculated using the geometric surface of the working electrode that was exposed to the electrolyte solution. The cyclic voltammograms shown in the article correspond to typical results obtained under the same experimental conditions.

### 2.7 Formate Quantification

Production of formate by the *Dv*H-FDH cathode during chronoamperometric measurements was quantified using an ionic chromatography-conductivity detector (Metrosep A Supp 7–250 × 4.0 mm column) in a Metrohm 883 Basic IC Plus using 3.6 mM sodium carbonate in water as the eluent (0.6 mL min^–1^). The chromatograph was also equipped with a Metrohm Suppressor Module (MSM) with 250 mM H_2_SO_4_ and 100 mM oxalic acid as the regenerating solution and 5% acetone in type 1 water as the rinsing solution. The calibration curve was obtained under electrolyte cell conditions and the formate was identified by the presence of the peak at the retention time of 8.206 min after ultrafiltration through a 0.22 μm RC filter.

### 2.8 AFM Measurements

Gold-coated slides from Arrandee Metal GmbH were first cleaned for 10 min in piranha solution and later flame-annealed in order to obtain large and well-ordered Au(111) terraces. Immobilization of *Dv*H-FDH was carried out as described above for the polycrystalline gold electrodes.

Tapping mode AFM images were recorded with an atomic force microscope from Agilent Technologies 5500 with the substrate immersed in the same electrolyte in which the electrochemical measurements were done. All images were recorded at room temperature using silicon nitrile cantilevers with a nominal spring constant of 0.72 N/m and a resonance frequency of 70 KHz (Olympus OMCL-RC). PicoView 1.3 (Agilent Technologies) and WSxM 5.0 Develop 8.0 (NanoTech) were employed for data acquisition and analysis, respectively.

### 2.9 QCM Measurements

A QCM able to monitor both the frequency and dissipation changes (KSV QCM-Z500) was employed to quantify the amount of *Dv*H-FDH covalently bound to a gold substrate modified with a SAM of 4-ATP. The frequency change (Δ*f*) was directly transformed into surface-adsorbed mass using the Sauerbrey equation.^21^ Previous to each experiment, gold-coated QCM crystal sensors with 5 MHz fundamental resonance frequency (QSense QSX 301) were properly cleaned and activated. Gold substrates were cleaned with a piranha solution for 5 min, rinsed with MilliQ water, and dried under nitrogen prior to the formation of a thiol SAM by exposing the surface to a 1 mM solution of 4-ATP in ethanol overnight at room temperature. Finally, gold substrates were rinsed with ETOH and then with MilliQ water. After that, the modified QCM crystal was immersed in a 10 mM MES solution pH 6 for 1 h to protonate the amino group on the surface and finally was dried under nitrogen before mounting in the QCM chamber. All measurements were performed at an operating frequency of 35 MHz (i.e., seventh overtone), with a continuous flow rate of 50 μL min^–1^ and a fixed temperature of 25 °C.

## 3 Results

### 3.1 Covalent Immobilization of *Dv*H-FDH on Gold Substrates

Two types of gold surface modifications were tested in order to expose amino groups. Positively charged amino groups on the surface should allow electrostatic orientation of *Dv*H-FDH molecules upon their deposition with the negatively charged region surrounding the most exposed Fe_4_S_4_ cluster facing the electrode (Figure 1A). In the first method, a 4-ATP SAM monolayer is formed on the gold surface, allowing the formation of amide bonds with the enzyme’s carboxylic residues surrounding the exposed Fe_4_S_4_ cluster (Figure 1B). In the second method, aminophenyl (AP) groups on the gold surface are formed by electrochemical reduction of a diazonium salt derivative and subsequently the non-modified gold regions are blocked by self-assembling MH (Figure 1C).

**Figure 2:**
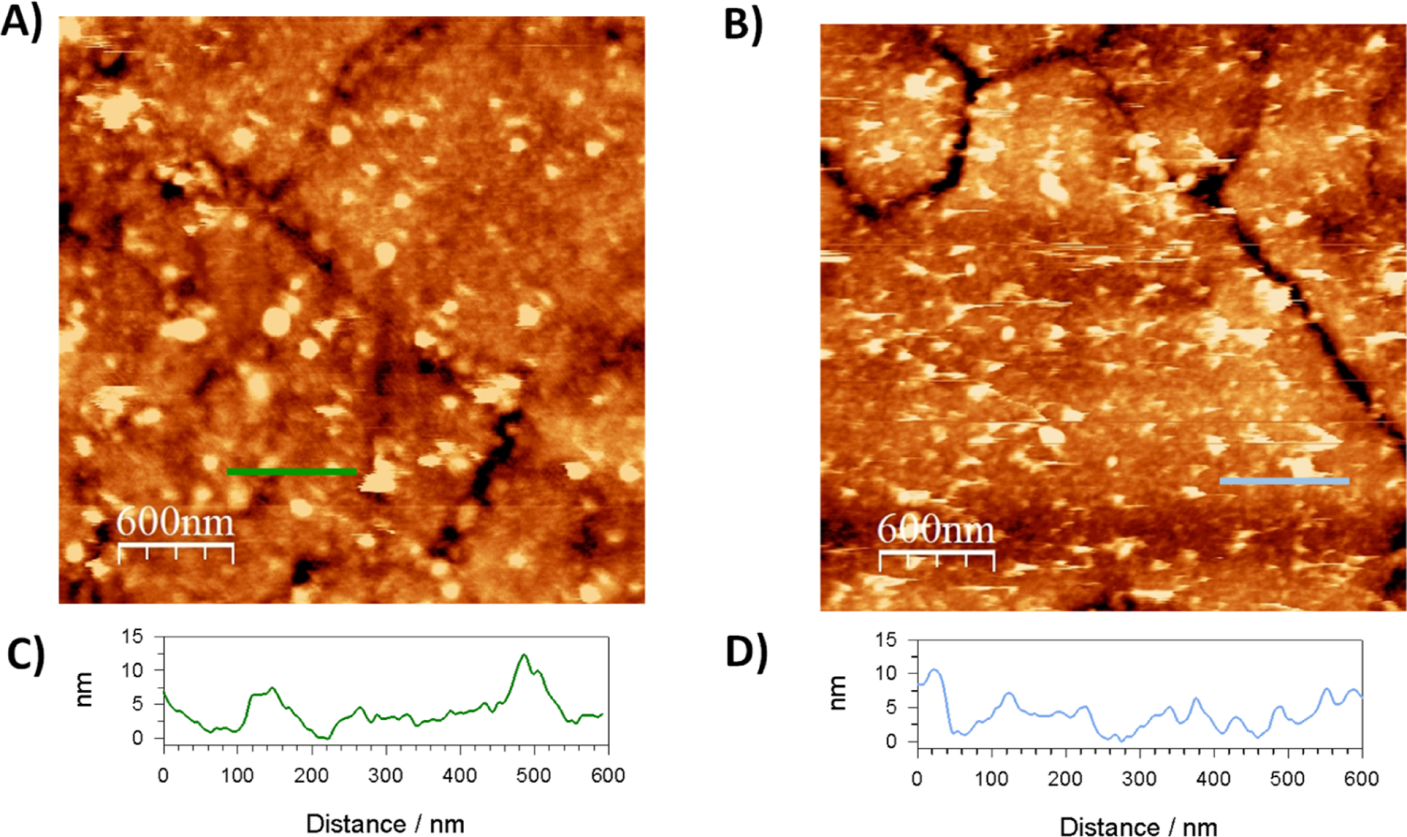
AFM topography images of *Dv*H-FDH covalently immobilized onto gold substrates modified with (A) 4-ATP SAM and (B) mixed AP + MH layers. (C,D) Cross-sectional domains of A and B, respectively.

The enzyme-modified gold surfaces were imaged by AFM. Figure 2 shows images corresponding to *Dv*H-FDH immobilized on different monolayers, one with a 4-ATP SAM (A) and the other with a mixed AP + MH layer (B). The presence of covalently bound *Dv*H-FDH changed the appearance of the gold surface. Control images of the surfaces without enzyme are shown in Figure S1, comprising the bare clean gold, the gold covered with a 4-ATP monolayer, and the gold covered with AP, as well as the gold covered with the AP + MH mixed layer. Without the enzyme, the roughness is below 5 nm and the grooves separating the terraces are well defined. The aggregates decorating the edges of the monolayer with 4-ATP are possibly due to the preference of the molecules to adsorb onto the more reactive edges of the gold terraces, as has been described previously.^22^ In contrast, the gold terraces modified with the enzyme show increased roughness, while the clefts between them were smoothed and compatible with protein molecules being deposited inside the grooves (Figure 2). Qualitatively, the appearance is that of a compact protein layer with the presence of some higher protuberances of 20 nm, which could correspond to a few protein molecules aggregated (Figure S2).

**Figure 3:**
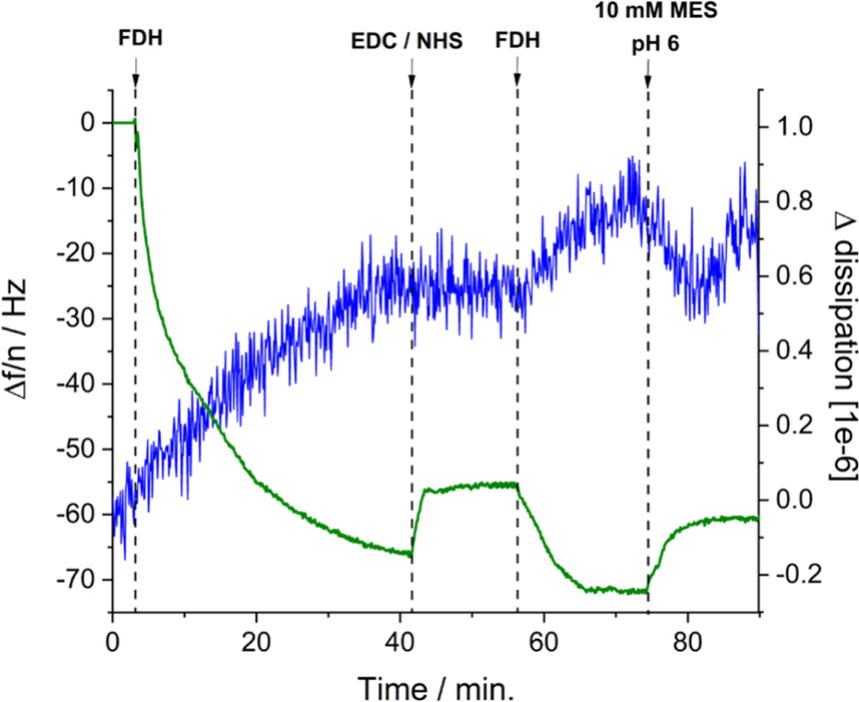
QCM frequency (solid green line) and dissipation (solid blue line) response (seventh overtone) for the covalent immobilization process of *Dv*H-FDH on gold substrates modified with 4-ATP SAM.

In order to quantify the amount of protein attached using this immobilization protocol, we proceeded to perform QCM experiments. This technique allows associating the changes in the resonance frequency of a piezoelectric quartz crystal to the amount of mass deposited on its surface. We modified the gold surface on the quartz crystal with a 4-ATP monolayer, immersed it for 1 h in a pH 6 10 mM MES solution to protonate the amino groups and placed it on the equipment. Afterward, we monitored the changes in the resonance frequency as we flowed over the surface different solutions. Figure 3 shows the changes in the frequency (left axis) and in the dissipation (right axis) registered as a function of time. Black arrows show the time at which each solution was injected into the measuring chamber. Flowing a 10 mM MES buffer pH 6.0 solution containing 1 μM *Dv*H-FDH during approximately 30 min yielded a 66 Hz change in the signal. Then, we substituted the solution with 10 mM MES pH 6.0 buffer containing 7.7 mM EDC and 9.5 mM NHS instead of the enzyme, aiming to activate the formation of the covalent link between the exposed carboxylic groups of the enzyme and the amino groups available in the 4-ATP SAM. The 10 Hz increase of the frequency could be due to slight changes in the viscosity of the medium or to a small release of the adsorbed enzyme. Afterward, we injected the *Dv*H-FDH fresh solution again to check if the activation induced further crosslinking between the flowing enzyme and the FDH is attached to the surface. We observed a reversible decrease of only a few Hz, which increased again when the surface was rinsed with a buffer solution. This sensogram indicates that the enzyme covalently attached to the surface was the one adsorbed initially due to the electrostatic interaction between the negatively charged carboxyl groups of *Dv*H-FDH and the positively charged amino groups on the surface. Addition of EDC stabilized the enzyme layer to further rinsing. The changes in the dissipation during the whole process remained below 1 × 10^–6^, indicating that the material adsorbed onto the surface was firmly packed and therefore the Sauerbrey equation can be used to associate the frequency changes observed to the mass of enzyme deposited on the surface. This calculation determines that the enzyme coverage was (8.6 ± 0.2) × 10^–12^ mol cm^–2^.

### 3.2 Bioelectrocatalytic Oxidation of Formate to Carbon Dioxide with Modified Gold Electrodes

Both the AFM and QCM measurements confirmed that *Dv*H-FDH was covalently anchored to the gold substrates. In order to verify that the immobilized enzyme retained its catalytic activity and also was oriented with its exposed redox center close to the gold surface, we studied the bioelectrocatalytic oxidation of formate to CO_2_ by DET for both surface-modification strategies. However, these two strategies did not allow studying the reverse activity of CO_2_ reduction to formate because the reductive desorption of assembled thiols starts at −0.39 V versus SHE in pH 7.6 phosphate buffer (Figure S3), which is higher than the thermodynamic value for reduction of CO_2_.^23^

**Figure 4:**
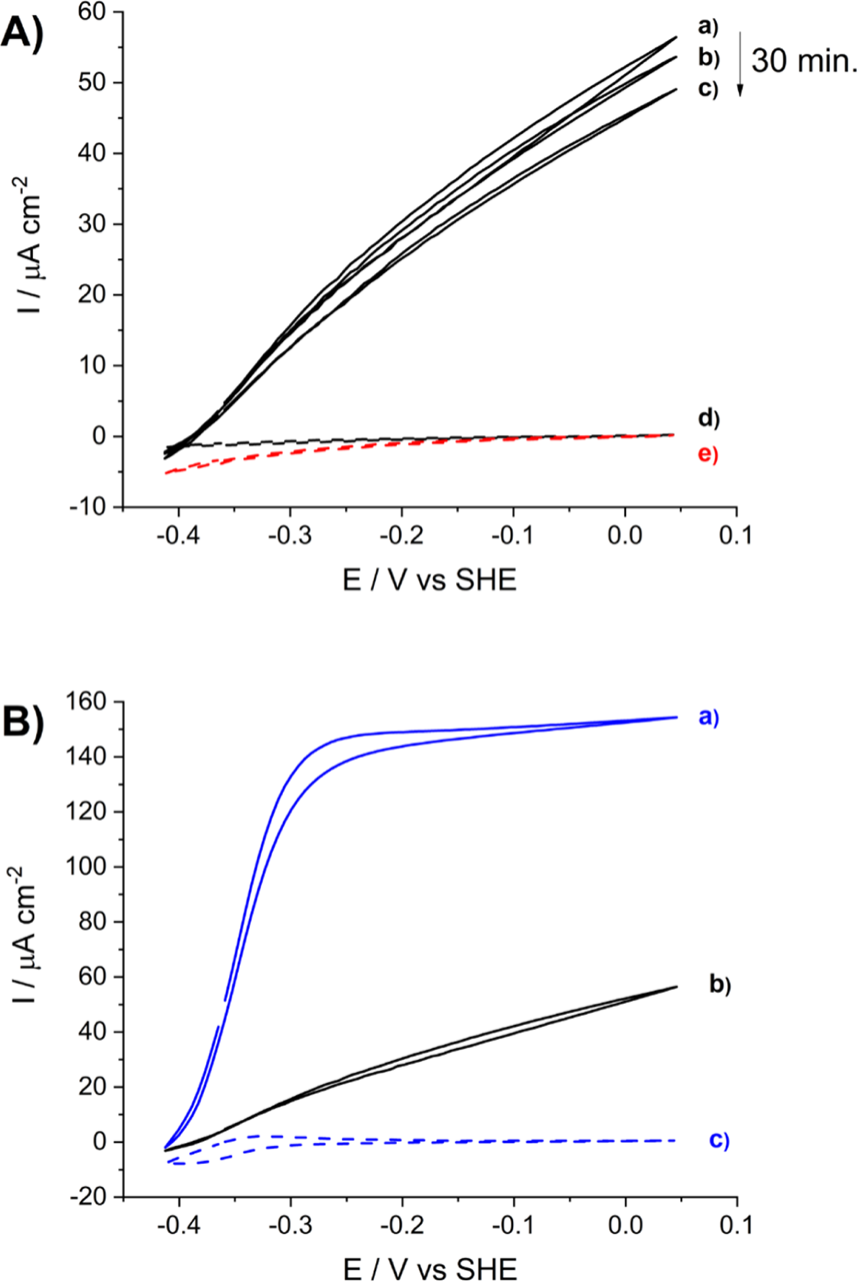
Bioelectrocatalytic oxidation of 20 mM formate in 0.1 M phosphate, pH 7.6 buffer measured for *Dv*H-FDH covalently immobilized on gold electrodes modified with 4-ATP SAM. (A) Consecutive voltammetry cycles in the DET regime for 30 min (black solid lines, a–c); the dashed black (d) and red (e) voltammograms correspond to the control electrodes without the immobilized enzyme and without formate, respectively. (B) Comparison of MET- and DET-based bioelectrocatalysis: cyclic voltammograms in the presence (solid blue line, a) and absence (black solid line, b) of 0.16 mM benzyl viologen; the dashed blue voltammogram (c) corresponds to the control electrode without the immobilized enzyme and in the presence of 0.16 mM benzyl viologen. Scan rate was 0.01 V s^–1^ and the temperature was 25 °C.

Figure 4 shows the electrochemical response of *Dv*H-FDH covalently bound to gold electrodes modified with 4-ATP SAM amino groups in the presence of 20 mM formate. Cyclic voltammograms in the absence of a redox mediator were recorded during 30 min for measuring the oxidation of formate at the Au/4-ATP/FDH electrode by the DET regime (Figure 4A, a–c). It can be observed that the electroactivity of D*v*H-FDH gradually decreased with successive cycles. However, a comparison with the voltammograms of the control electrodes lacking either FDH (Figure 4A, d) or formate (Figure 4A, e) indicates that the signal loss is only 15% (at the most positive potential) after 30 min of sweeping. This result suggests either partial enzyme desorption, inhibition, or denaturation with time. The onset of electrocatalytic current is at −0.410 V, which is approximately at the thermodynamic value of the CO_2_/formate pair and in agreement with a reversible catalyst immobilized on the electrode.^23^ Furthermore, the shape of the catalytic wave that does not reach a plateau and increases almost linearly with the overpotential is typical of protein film voltammetry rate-limited by the DET step.^14,17,23,24^

In order to reveal the full electroactivity of the enzyme molecules immobilized on the electrode, we added the redox mediator benzyl viologen to the solution. In this case, mediated electron transfer (MET) bioelectrocatalysis led to a sigmoidal cyclic voltammogram reaching a current plateau at just 0.1 V overpotential. Moreover, the MET-based catalytic current was approximately 3 times larger than the DET-based one at the highest overpotential measured (Figure 4B, a). These characteristics of the cyclic voltammogram under quiescent conditions clearly suggest an electrocatalytic process rate-limited by the enzymatic reaction.^24^

**Figure 5:**
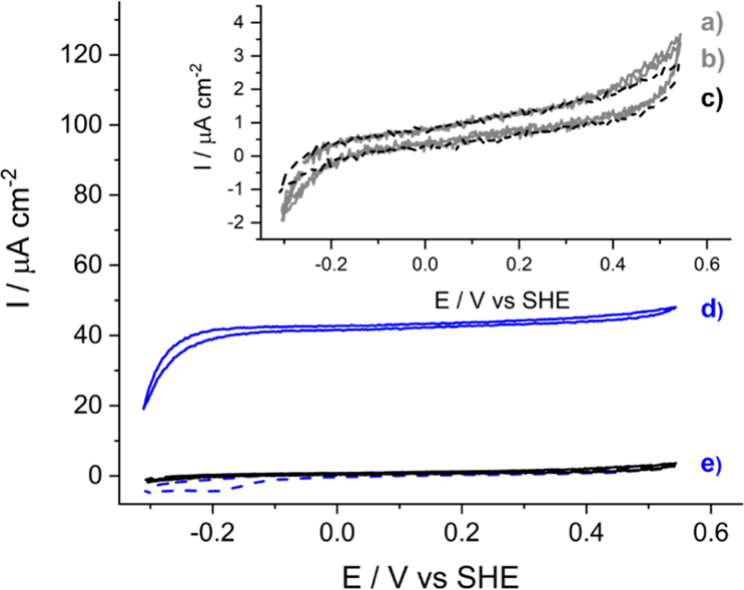
Bioelectrocatalytic oxidation of 20 mM formate, 0.1 M phosphate, pH 7.6 buffer measured for *Dv*H-FDH covalently immobilized on gold electrodes modified with a mixed AP + MH layer. Cyclic voltammograms measured in the absence (solid gray lines, a,b) and in the presence (solid blue line, d) of 0.16 mM BV. The dashed black, c, and blue, e, voltammograms correspond to the control electrode without the immobilized enzyme in the absence and in the presence of the redox mediator, respectively. The scan rate was 0.01 V s^–1^ and the temperature was 25 °C. Inset: magnification of the cyclic voltammograms in the absence of BV.

On the other hand, when *Dv*H-FDH was immobilized on a gold electrode modified with a mixed AP + MH layer, the DET-based electrocatalytic current was almost negligible and observed only at a very high overpotential (Figure 5, a,b inset). This result clearly demonstrates that the DET between the enzyme and the electrode is inefficient when *Dv*H-FDH is covalently bound to the mixed AP + MH layer. Nevertheless, a clear MET-based electrocatalytic process was observed in the presence of benzyl viologen in solution, although the catalytic plateau current (approximately 30 μA cm^–2^, Figure 5) was 4 times lower in comparison with the result obtained by immobilization of the enzyme on the 4-ATP SAM (approximately 120 μA cm^–2^, Figure 4B, a). By increasing the amount of *Dv*H-FDH during the immobilization onto the gold electrode modified with the mixed AP + MH layer, the MET-based bioelectrocatalytic plateau current increased, although the DET-based process did not improve (Figure S4). Thus, we discard this immobilization method as a valid one for optimizing the DET of *Dv*H-FDH on gold, most probably because it does not lead to a correct orientation of the W-FDH with its most exposed Fe_4_S_4_ cluster near the electrode surface.

### 3.3 Bioelectrocatalytic Reduction of Carbon Dioxide to Formate with Graphite Electrodes

*Dv*H-FDH is an ideal model system for electrocatalytic studies of CO_2_ reduction because it has been found to be the most active enzyme for this process.^13^ As we indicated above, the negative potentials needed for CO_2_ reduction are not compatible with the stability of thiols’ SAM used for the covalent immobilization of the enzyme on gold electrodes. Aiming to overcome this limitation, we have developed a strategy for covalent bonding of *Dv*H-FDH that allows reaching the necessary negative potentials. The functionalization of LDG electrodes with an AP layer by diazonium reduction is a valid alternative because it avoids the incorporation of the MH SAM required to prevent the enzyme denaturalization that occurs on bare gold. First, we verified that this method led to the immobilization of *Dv*H-FDH in an oriented fashion that allows the electrocatalytic activity for formate reduction by DET. Figure S5 shows that the measured catalytic currents for formate oxidation, with just 0.1 V of overpotential, are higher with modified LDG electrodes, either in the DET (322 μA cm^–2^) or MET (1180 μA cm^–2^) regime, compared to gold electrodes. This can be attributed to the much higher porosity of the LDG electrodes that allows higher loading of the immobilized enzyme.^25^ The cyclic voltammogram recorded in the presence of the substrate in the MET regime yielded a peaked shape instead of the sigmoidal shape expected for an electrocatalytic process. This effect is attributed to the high catalytic current, which leads to mass-transfer limitation toward the electrode surface in a quiescent solution. Indeed, the increase of the mass transport rate of the substrate and redox mediator by electrode rotation led to a significant increase of the catalytic current (Figure S6). On the other hand, the DET-based electrocatalytic current barely increased upon electrode rotation, confirming that in that case interfacial electron transfer from the immobilized *Dv*H-FDH to the electrode was the rate-limiting step.

**Figure 6:**
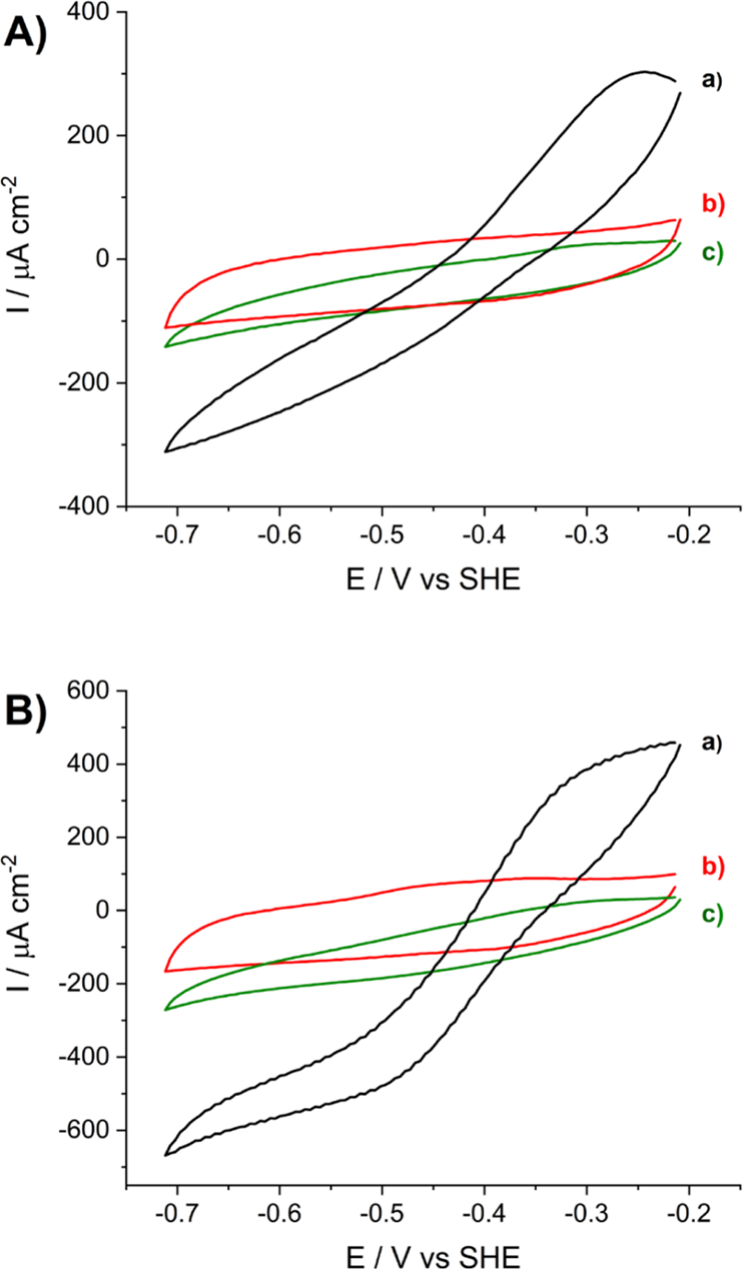
Bioelectrocatalytic reduction of CO_2_ measured for *Dv*H-FDH covalently immobilized on a LDG/AP electrode in 0.1 M citrate buffer at pH 6.0 containing 50 mM NaHCO_3_. Cyclic voltammograms in (A) DET and (B) MET regime (0.16 mM methyl viologen) before (green solid lines, c) and after (black solid lines, a) the addition of 50 mM NaHCO_3_. The CV curves of the control electrodes without immobilized *Dv*H-FDH and in the presence of 50 mM NaHCO_3_ are shown as red lines, b. The scan rate was 0.01 V s^–1^ and the temperature was 25 °C.

Once it was checked that the *Dv*H-FDH covalently immobilized on LDG electrodes was active for formate oxidation, the reverse reaction of CO_2_ reduction was studied. As the CO_2_ reduction activity of FDH is considerably lower than the formate oxidation one,^6,13^ we doubled the amount of enzyme during the immobilization step. Figure 6 shows the cyclic voltammograms of the LDG/AP/FDH electrode in a pH 6.0 citrate buffer solution containing 50 mM NaHCO_3_ to obtain an excess of dissolved CO_2_ that does not limit the enzyme kinetics.^13,23^ Under the DET regime, the cyclic voltammograms in Figure 6A show the immobilized *Dv*H-FDH catalyzing the interconversion between CO_2_ and formate, which was generated at the electrode surface at the lower potentials, close to the thermodynamic potential. Moreover, the peaked shape of the cyclic voltammogram was probably due to depletion of formate at the electrode surroundings when higher potentials caused *Dv*H-FDH formate-oxidizing activity. Under the MET regime in the presence of methyl viologen (Figure 4B), the enzyme activity increased 3-fold, in agreement with the experiments performed in the presence of formate (Figure S5).

**Figure 7:**
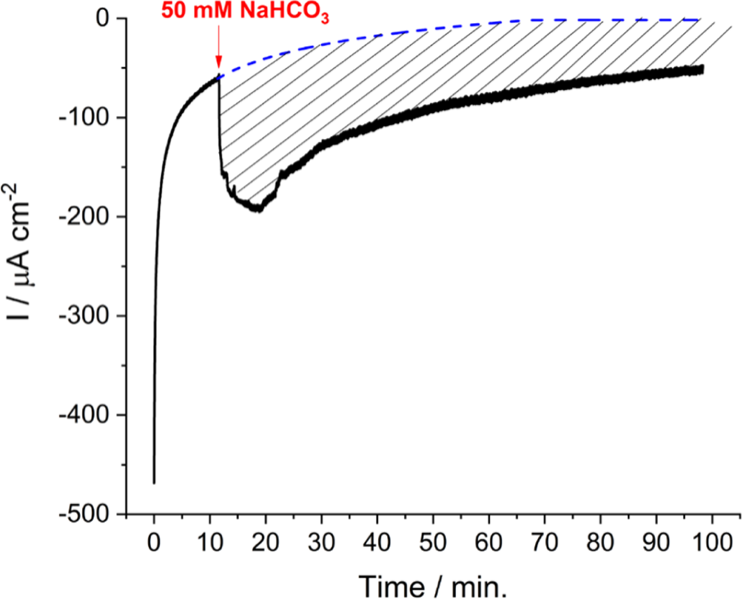
Chronoamperometry at −0.66 V vs SHE under stirring with a LDG/AP/FDH electrode in the presence of 50 mM NaHCO_3_ in 0.1 M citrate buffer at pH 6.0 and 25 °C. The red arrow indicates substrate addition and the dashed blue line represents the projection of the background current in the absence of substrate for integrating the charge of the catalytic process.

The operational stability of the FHD immobilized on the LDG electrode for electrocatalytic CO_2_ reduction by DET was studied by choronoamperometry at −0.66 V versus SHE (Figure 7). After the addition of 50 mM sodium bicarbonate, the current density reached a value of −200 μA cm^–2^. The catalytic current density decreased gradually with time, although the electrode showed an operational stability of approximately 90 min, at which 34% of the initial catalytic current was maintained (Figure 7). Integration of the total charge produced during several chronamperometric measurements allowed estimating the concentration of produced formate assuming a 100% Faradaic yield of the bioelectrocatalytic process. The average value from three measurements done with different *Dv*H-FDH-LDG electrodes was 3.3 ± 0.4 μM. The negative control measurement was done by a previous immersion of the *Dv*H-FDH-LDG electrode in 2 M HCl for 5 min in order to denature the enzyme, which was then thoroughly washed in 10 mM MES, pH 6.0 buffer before measuring the chronoamperometry in the presence of 50 mM NaHCO_3_. The chronoamperometry obtained with the denatured electrode yielded the background current signal (Figure S7).

We studied the effect of crosslinking the enzymes using 0.9% glutaraldehyde after the covalent immobilization on LDG electrodes with the aim of increasing the operational stability for the bioelectrocatalytic reduction of CO_2_. The crosslinking with glutaraldehyde during 60 min caused a remarkable decrease in the activity of the enzyme, as the electrocatalytic current was reduced more than 50%. The crosslinking for 30 min increased the stability of the immobilized *Dv*H-FDH, improving the operational time up to approximately 3.5 h (Figure S8). However, the calculated amount of CO_2_ reduced to formate from the chronoamperometric measurement was similar to the measurements performed without crosslinking the enzyme (Table S1), due to the initial decrease of the electrocatalytic current caused by such crosslinking process. For that reason, the crosslinking step with glutaraldehyde was forsaken.

**Figure 8:**
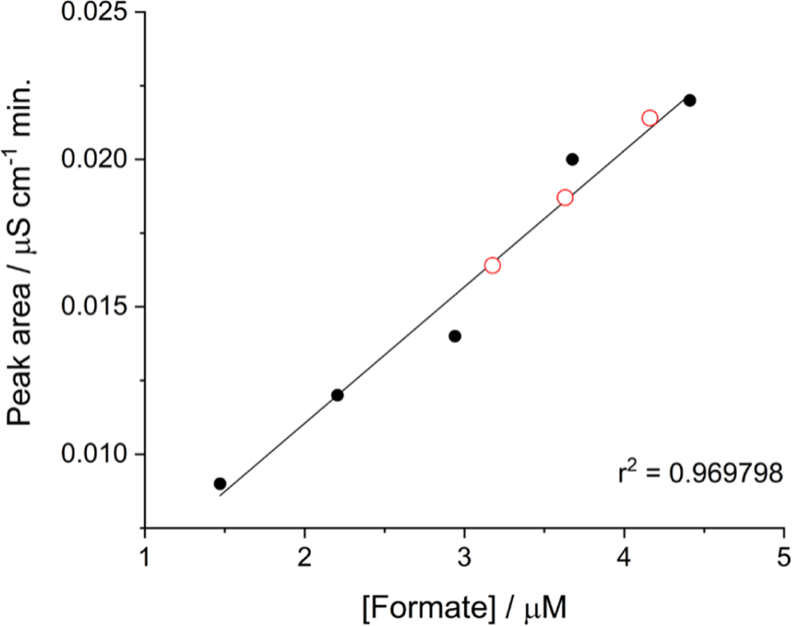
Quantification by ionic chromatography of formate concentration produced with a LDG-AP-FDH electrode during typical chronoamperometry curves at −0.66 V vs SHE under stirring in the presence of 50 mM NaHCO_3_ in 0.1 M citrate buffer at pH 6.0 and 25 °C. The black circles correspond to the formate calibration plot and the red open red circles correspond to the three electrolyte samples taken from the chronoamperometric measurement.

Formate production was also quantified by ionic chromatography. The calibration used for formate determination by ionic chromatography and the values for three replicates from chronoamperometric measurements are shown in Figure 8. The average concentration of formate produced after 90 min of chronoamperometry was 3.7 ± 0.5 μM, a very similar value to that estimated from charge integration taking into account the experimental error.

## 4 Discussion

In this work, we have studied two different strategies aimed to obtain oriented covalent immobilization of *Dv*H-FDH on either gold or graphite electrodes with its most exposed redox site facing the electrode surface. Even if the thiol-modified gold electrode strategy only allows studying the formate oxidation activity of *Dv*H-FDH and not the CO_2_ reduction one, it has the advantage of allowing the characterization of the modified electrode by AFM and QCM. Therefore, the FDH-modified electrode conformation can be correlated with its catalytic function.

Both the formation of a 4-ATP SAM over gold and the functionalization of LDG with aminophenyl groups by electrochemical reduction of a diazonium derivative have been suitable for measuring high electrocatalytic currents of formate oxidation by DET. The results are comparable to those previously reported using the same strategies for *Desulfovibrio gigas* NiFe-hydrogenase, which has a similar charge distribution in the protein surface.^17,26^ Therefore, we confirm that electrostatic interactions between the positively charged amino groups on the electrode surface and the negatively charged amino acid residues surrounding the most exposed Fe_4_S_4_ cluster modulate the orientation of an enzyme monolayer previous to the covalent immobilization step. However, a distinctive behavior is observed for the immobilized *Dv*H-FDH when a redox mediator is added to the solution. The MET-based electrocatalytic current densities increased 3–4 times that of the DET-based ones (depending on the overpotential at which they are measured) with both Au/4-ATP and LDG/AP electrodes, whereas in our previous works of oriented covalent immobilization of hydrogenases and laccases on electrodes, the addition of a redox mediator in solution seldom increased the DET-based electrocatalytic currents.^19,26^ The AFM and QCM characterization performed in the present work for the *Dv*H-FDH covalently bound to Au/4-ATP electrodes provides an explanation for this discrepancy. The AFM images suggest a compact layer of protein attached to the modified gold surface including numerous aggregates with heights of 15–20 nm, which can be assigned to two or three crosslinked enzyme molecules. In contrast, topographic profiles obtained from AFM images of *D. gigas* NiFe-hydrogenase,^17^*D. vulgaris* NiFeSe-hydrogenase (*Dv*H-Hase)^27^ and *Trametes hirsuta* laccase^19^ covalently bound on functionalized gold surfaces were compatible with a more spaced monolayer of attached enzyme molecules. The QCM study of the *Dv*H-FDH covalent binding to Au/4-ATP has allowed determining the enzyme coverage, (8.6 ± 0.2) × 10^–12^ mol cm^–2^, which is approximately 2 to 3 times higher than the value expected for a spaced monolayer of an enzyme of this dimension. Therefore, both QCM and AFM measurements point to the presence of crosslinked enzyme molecules on top of the monolayer directly bound to the surface, which are too far to participate on DET with the electrode but are capable of MET-based electrocatalysis. Indeed, the crystal structures of *Dv*H-FDH^13^ and *Dv*H-Hase^28^ indicate a much higher amount of lysine residues in the protein surface of the former than in the latter, which could favor crosslinking during the carbodiimide coupling step of the immobilization process.

The bioelectrocatalytic currents measured for formate oxidation, either by DET or MET, are much higher when the *Dv*H-FDH was covalently immobilized onto the LDG/AP electrodes than onto the Au/4-ATP. These results are not surprising as the electroactive area of the former, a highly porous material,^25^ is much larger than that of planar gold. Thus, a higher coverage of immobilized enzyme per geometric surface of electrode is expected in the first case. For technical reasons, we could not directly determine the enzyme loading on LDG electrodes by QCM, although we can estimate this value from the ratio between the two types of electrodes of plateau currents obtained in the presence of BV as a redox mediator. Under these conditions, the enzymatic reaction rate-limits the electrocatalytic process and we can assume that the current density value is proportional to the total amount of enzyme immobilized, as in both cases, it is covalently bound by the same chemical reaction. In this way, we can estimate an enzyme coverage of 1.06 × 10^–10^ mol cm^–2^ on the modified LDG electrodes.

The covalent immobilization of *Dv*H-FDH onto LDG/AP electrodes also allowed measuring the electrocatalytic currents due to the reverse activity of CO_2_ reduction, which is of more interest from the technological point of view. In similar way to the formate oxidation electroactivity, the MET-based electrocatalytic current was significantly higher than the DET-based one. For comparison of data obtained with electrodes with different electroactive areas and enzyme loadings, it is useful to calculate the apparent catalytic rate (*k*_cat, app_) of the immobilized enzyme normalizing the electrocatalytic current densities at a certain overpotential by the enzyme coverage, and taking into account that the conversion between formate and CO_2_ involves two electrons. In this way, in the case of MET-based electrocatalysis, we obtain from the catalytic plateau currents (thus independent of the potential) a *k*_cat,app_ of 90.6 and 8.6 s^–1^ for formate oxidation and CO_2_ reduction, respectively. These results confirm the bias of FDH toward the formate oxidation direction of their native catalytic activity measured in solution by spectrophotometry.^6,13^ In the case of the DET mechanism, we obtain values of *k*_cat,app_ (at 0.24 V overpotential relative to the thermodynamic value at the measured pH) of 20.5 and 10.2 s^–1^ for formate oxidation on Au/4-ATP and LDG/AP, respectively, whereas for CO_2_ reduction on LDG/AP, it is 3.5 s^–1^. In the DET mode, the measured CV curves reflect that the electrocatalytic currents are mostly limited by the interfacial electron transfer of the enzyme with the electrode, thus the *k*_cat,app_ values for LDG/AP/FDH suggest an approximately 3 times faster rate in the oxidation direction than in the reduction direction. This result could be explained by a formal redox potential of the exposed Fe_4_S_4_ cluster of *Dv*FDH that is more negative than that of the formate/carbon dioxide pair.

There a several reports in the literature of FDH immobilization on electrodes, especially for studying their ability for electrocatalytic reduction CO_2_. A comparison of results is not straightforward as they involve different types of enzymes, electrodes, immobilization methods, and measurement conditions (pH, temperature). Nevertheless, it is of interest to do a comparative study in the case of NADH-independent FDHs, that is, both Mo- and W-FDHs, which share common characteristics and have been immobilized on electrodes by different methods. In most studies reported in the literature, the metal containing FDHs have been either directly deposited on the electrode surface by adsorption,^14,23,29−31^ crosslinked with glutaraldehyde,^32−34^ or entrapped within a redox polymer,^15,35^ whereas covalent bonding to the functionalized surface has seldom been studied. Higher electrocatalytic currents of CO_2_ reduction are obtained with electrodes with very large electroactive areas and that allow fast mass transport of the substrate, such as carbon cloth gas-diffusion-type electrodes.^15,33^ In order to compare data from bioelectrodes with very different electroactive areas and enzyme loadings with those obtained in this work, it is useful to estimate their *k*_cat,app_ values, as shown in Table S2. The *k*_cat,app_ of 3.5 s^–1^ we obtained for DET-based CO_2_ reduction by *Dv*H-FDH on the LDG-modified electrode is almost of the same value estimated from the reported results for the same enzyme adsorbed onto *meso*TiO_2_,^14^ a mesoporous material shown to have strong and stable affinity to redox metalloenzymes while allowing fast DET. A 2-fold increase of the *k*_cat,app_ is determined for another W-FDH crosslinked to a carbon cloth electrode coated with a conductive polymer that serves as a wire for electron transfer to the enzyme.^34^ However, the highest *k*_cat,app_ of 91 s^–1^ was obtained for an adsorbed monolayer of a W-FDH on a planar electrode of highly oriented pyrolytic graphite edge, although the operational stability is very small due to continuous desorption of the enzyme, and 50% of the initial catalytic current is lost after 3.5 min of chronoamperometry.^23^ In the case of MET-based systems, higher *k*_cat,app_ for CO_2_ electroreduction are obtained with diffusing redox mediators in solution, such as in the present work, and as reported by Kano and co-workers,^33^ than with redox polymers,^15,35^ suggesting that electron hopping along the redox polymer to the redox site of the entrapped enzyme could be rate-limiting the electrocatalytic process. However, the addition of redox mediators in solution is not practical for applications, and the entrapment of FDH on these redox polymers affords the highest operational stabilities reported.^15,35^

We show in the present work that the covalent binding of *Dv*H-FDH to the LDG/AP electrode allowed measuring continuous reduction of CO_2_ to formate with approximately 100% Faradaic yield up to 2 h, although a constant decrease of the electrocatalytic current is evident. This is opposite to what we reported previously for hydrogenase covalently bound by the same strategy as carbon electrodes, in which the operational stability is reached in 1 month with hardly any current decrease.^26,36^ Therefore, it seems that *Dv*H-FDH is a more vulnerable enzyme to covalent modification, causing a lower operational stability.

## 5 Conclusions

The covalent binding of *Dv*H-FDH to Au/4-ATP and LDG/AP electrodes modulated by electrostatic orientations allows measuring efficient DET-based bioelectrocatalysis. AFM and QCM characterization of the modified gold electrodes, as well as a comparison of direct and mediated electrocatalysis, suggest that a compact layer of *Dv*H-FDH is anchored to the electrode surface with some crosslinked aggregates. Immobilization of *Dv*H-FDH on modified gold electrodes allows studying electrocatalytic oxidation of formate oxidation, although it does not allow further studies of electrocatalytic reduction of CO_2_ because reductive desorption of the assembled thiols appears at higher potential than the thermodynamic value for reduction of CO_2_. In contrast, *Dv*H-FDH covalently bound to LDG electrodes modified with the AP layer allows measuring high electrocatalytic currents by DET for both formate oxidation and carbon dioxide reduction, reaching values up to 700 and −200 μA cm^–2^, respectively. Chronoamperometric measurements at −0.6 V versus SHE during 90 min led to the production of 3.7 ± 0.5 μM formate with approximately 100% Faradaic yield. However, the operational stability was only about 90 min; crosslinking the enzyme with glutaraldehyde improved it to about 1.5 h more but decreased the initial electrocatalytic current density.

## Supporting Information Available

AFM images and topographic profiles of modified electrodes; cyclic voltammograms of thiol SAMs’ reductive desorption and bioelectrocatalytic oxidation of formate; chronoamperometry curves of bioelectrocatalytic oxidation of formate and CO_2_ reduction; integrated chronoamperometry data; and kinetic data for bioelectrocatalytic CO_2_ reduction (PDF)
