# Supplementary material for: Bioelectrocatalytic Activity of W-Formate Dehydrogenase Covalently Immobilized on Functionalized Gold and Graphite Electrodes
Source: ACS Appl Mater Interfaces. 2021 Mar 3;13(10):11891–900. doi: 10.1021/acsami.0c21932 (PMC8479727; doi:10.1021/acsami.0c21932)
Supplement: Supplementary file 1 — am0c21932_si_001.pdf [file am0c21932_si_001.pdf]

Supporting Information

Bioelectrocatalytic Activity of W-Formate Dehydrogenase Covalently Immobilized on Functionalized Gold and Graphite Electrodes

Julia Alvarez-Malmagro,[,a] Ana R. Oliveira,[b] Cristina Gutiérrez-Sánchez,[a] Beatriz Villajos,[a] Inês A.C. Pereira,[b] Marisela Vélez,[a] Marcos Pita,[a] Antonio L. De Lacey*[,a]*

[a]Instituto de Catálisis y Petroleoquímica, CSIC, c/Marie Curie 2, 28049 Madrid, Spain.

[b]Instituto de Tecnologia Química e Biologica, Universidade Nova de Lisboa, Apartado 127, 2781-901 Oeiras, Portugal.

*Corresponding authors: alopez@icp.csic.es and j.malmagro@csic.es

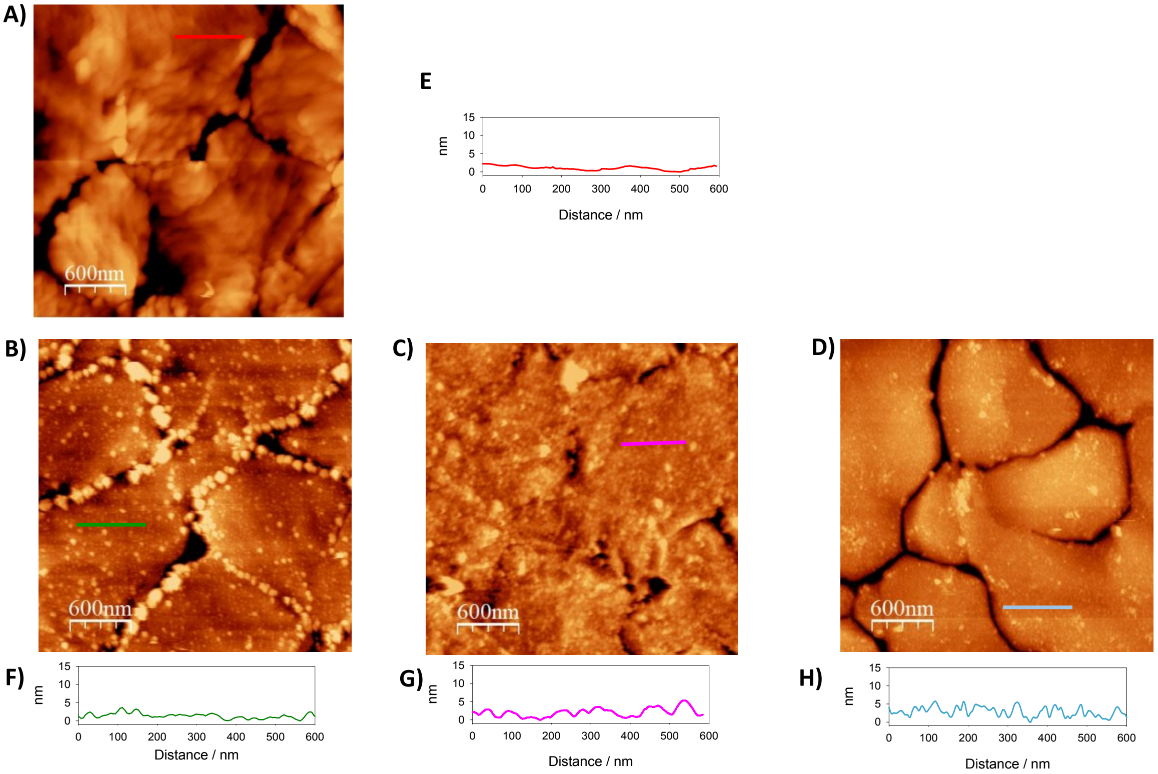

Figure S1. AFM topography images of A) a bare Au (111), B) a modified Au(111) surface with 4-ATP SAM C) a AP monolayer and D) a mixed AP/MH. (E,F,G,H). Cross-sectional domains of A, B, C and D respectively.

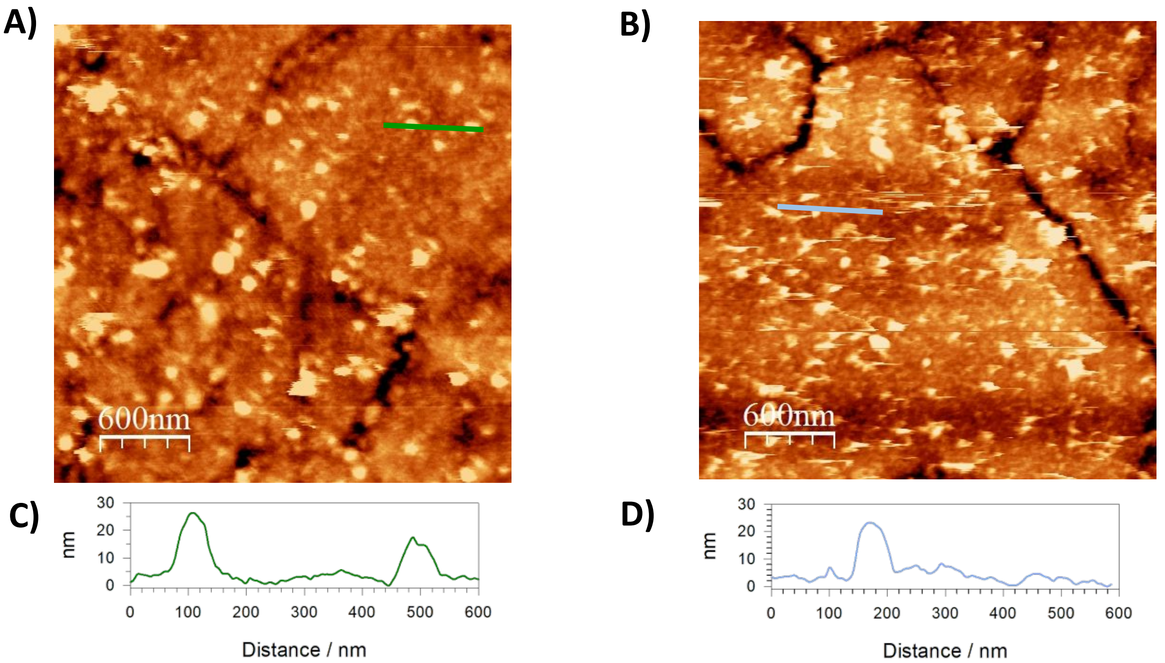

Figure S2. AFM topography images of *Dv*H-FDH covalently immobilized to gold substrates modified with A) 4-ATP SAM and B) mixed AP+MH layer. C, D) Cross-sectional domain of A and B, respectively.

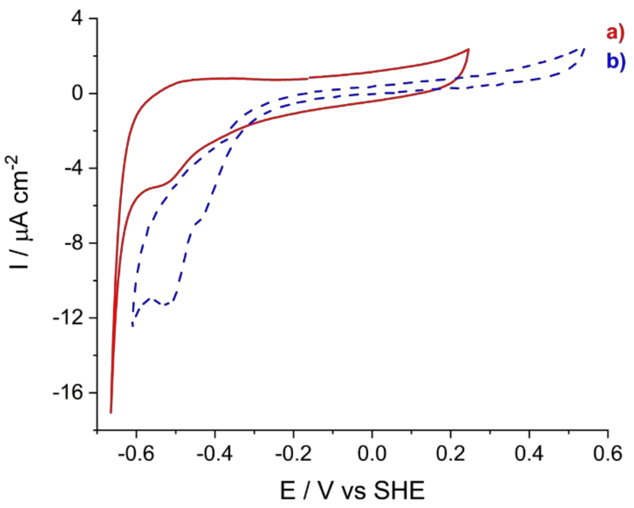

Figure S3. Cyclic voltammograms of the reductive desorption of a 4-ATP SAM (solid red line, a) and a mixed AP+MH layer (dashed blue line, b) on gold electrodes in 0.1 M phosphate buffer at pH 7.6. The scan rate was 0.01 V/s and the temperature was 25ºC.

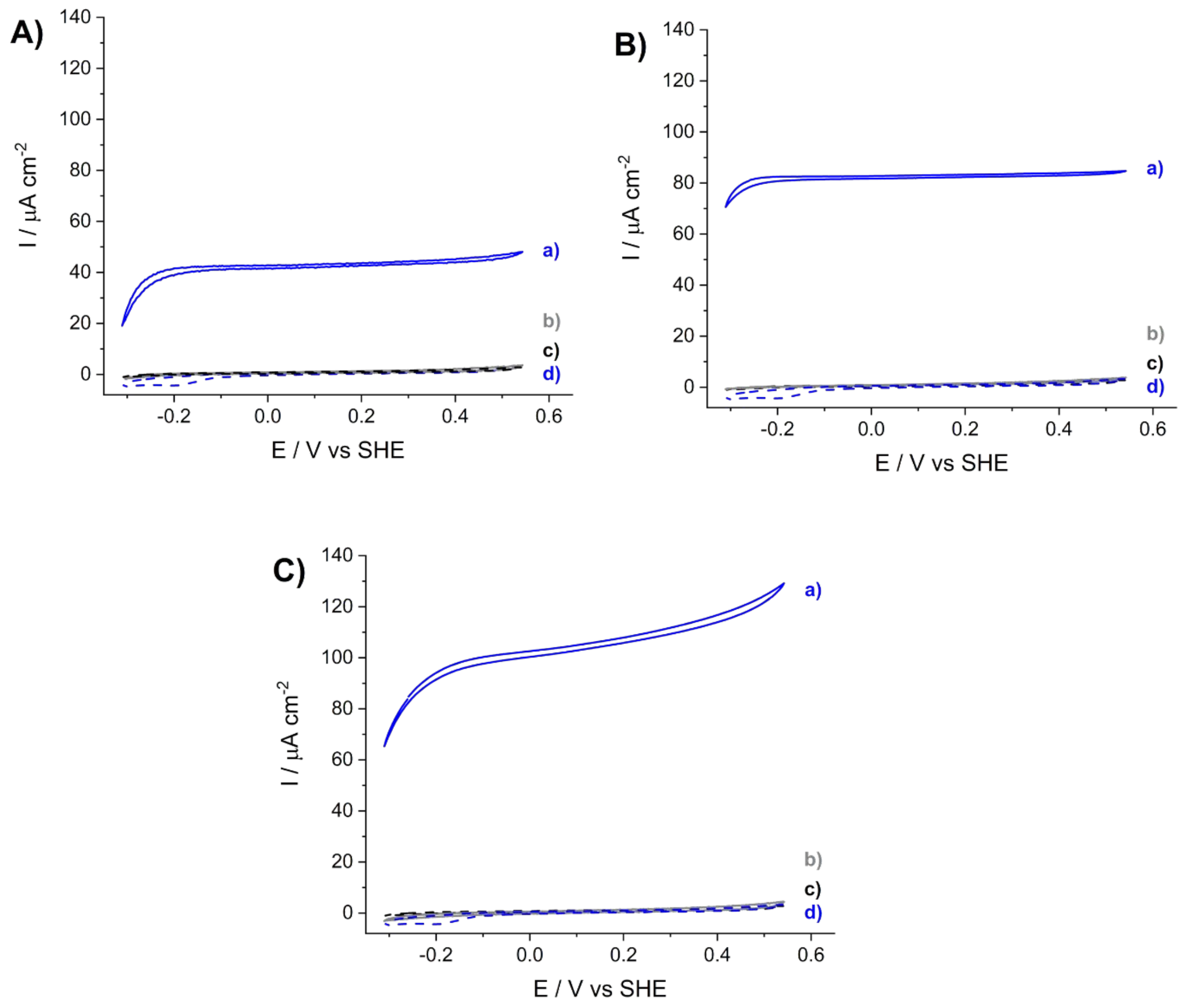

Figure S4. Bioelectrocatalytic oxidation of 20 mM formate 0.1 M phosphate, pH 7. 6 buffer measured for 8.1 (A), 16.2 (B) and 24.3 (C) μM *Dv*H-FDH covalently immobilized on gold electrodes modified with mixed AP/MH layer. Cyclic voltammograms measured in absence (solid grey line, b) and in presence (solid blue line, a) of 0.16 mM benzyl viologen. The dashed black, c and blue, d voltammograms correspond to the control electrode without immobilized enzyme in absence and in presence of redox mediator, respectively. Scan rate was 0.01 V/s and temperature was 25ºC.

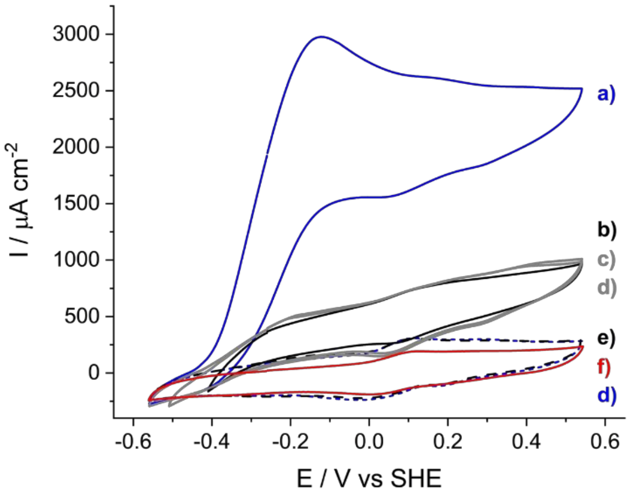

Figure S5. Bioelectrocatalytic oxidation of 20 mM formate in 0.1 M phosphate buffer at pH 7.6 and 25 °C measured for *Dv*H-FDH covalently immobilized on LDG electrodes modified with an AP layer. The first voltammetry cycle measured in DET mode is shown as black solid line, b, subsequent cycles for 30 minutes are in gray, c-d. The blue solid line, a, corresponds to the cyclic voltammogram measured in the presence of 0.16 mM benzyl viologen. The dashed black, e, and solid red, f, voltammograms correspond respectively to the control electrodes without immobilized enzyme and without formate respectively, and the dashed blue voltammogram, d, corresponds to the control electrode without immobilized enzyme and in presence of 0.16 mM benzyl viologen. Scan rate: 0.01 V s^{-1}.

Note: The oxidative and reductive peaks observed in the cyclic voltammograms between 0 and 0.2 V correspond to the redox process of the hydroxylamine groups present in the LDG surface.[1]

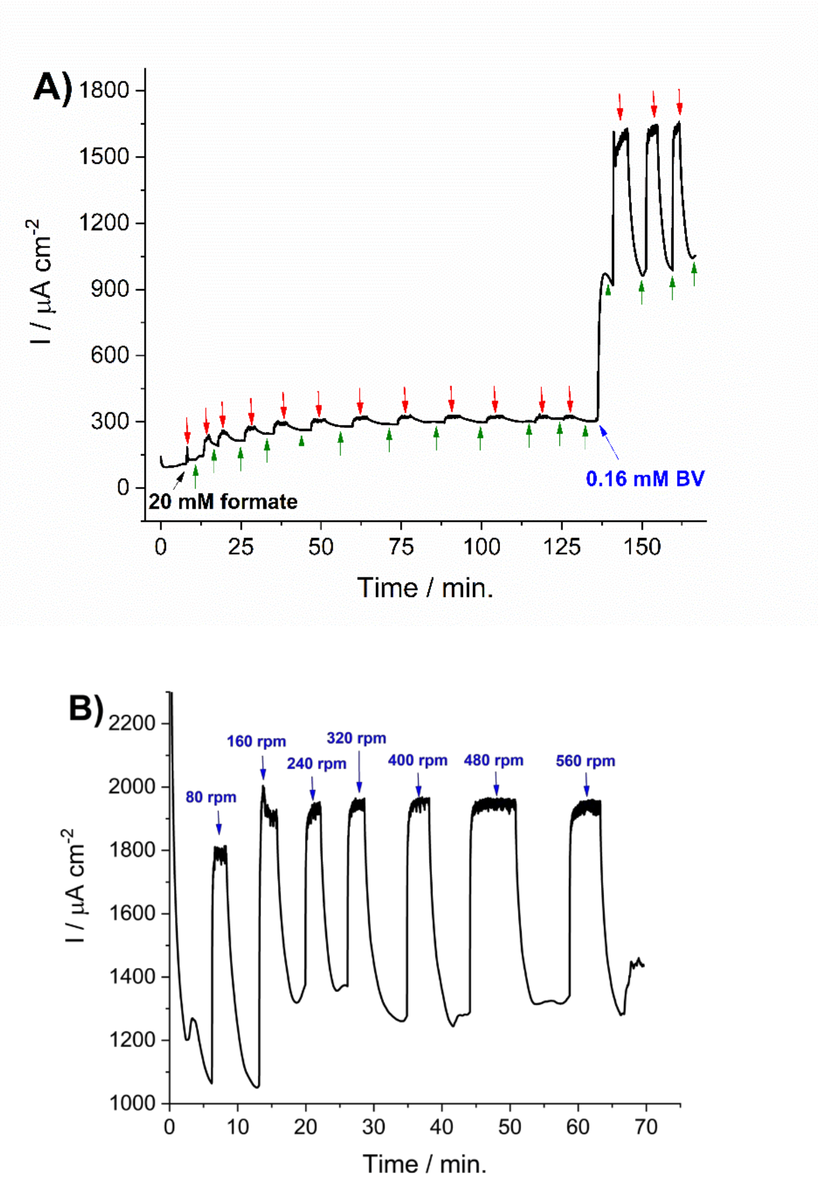

Figure S6. (A) Chronoamperometry at -0.06 V vs SHE measured for a LDG/AP/FDH electrode modified in 20 mM formate, 0.1 M phosphate buffer, pH 7.6, 25 °C. Red arrows indicate 80 rpm electrode rotation, green arrows indicate stationary solution, and the blue and black arrows indicate 20 mM formate and 0.16 mM BV addition respectively. (B) Chronoamperometry at -0.06 V vs SHE measured for a LDG/AP/FDH electrode in 20 mM formate in presence of 0.16 mM benzyl viologen at different rates of electrode rotation.

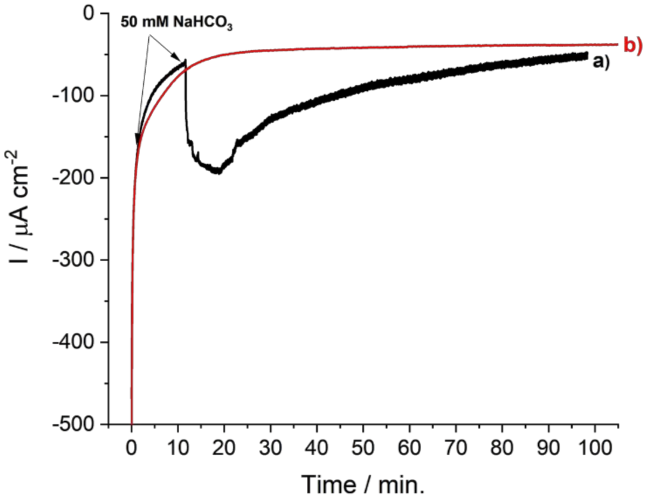

Figure S7. a) Chronoamperometry at -0.66 V vs SHE under stirring with a) a LDG-AP-FDH electrode (dark line) and b) LDG-AP-denaturalized FDH electrode (red line) in presence of 50 mM $NaHCO_3$ in 0.1 M citrate buffer at pH 6.0 and 25 °C.

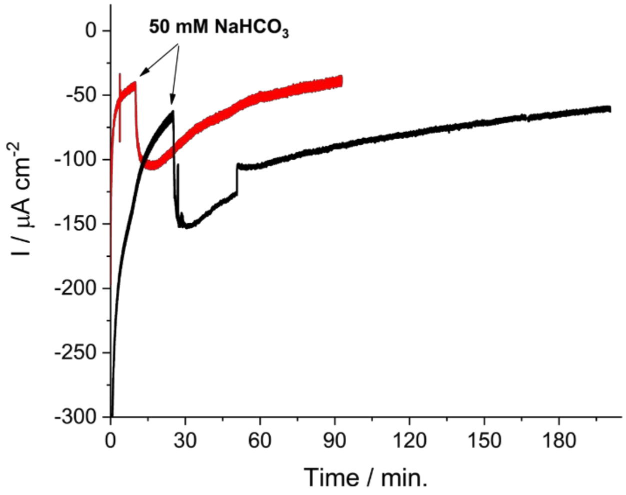

Figure S8. Chronoamperometry at -0.66 V vs SHE under stirring for reduction of CO_2 by FDH covalently immobilized on LDG/AP electrodes and crosslinked with 0.9 % glutaraldehyde for 60 (red solid line) and 30 minutes (black solid line). The electrolyte was 0.1 M acid citrate buffer at pH 6.0 and 25 °C.

Table S1. Formate concentration formed by reduction of CO_2 at LDG/AP/FDH electrodes estimated by charge integration of chronoamperometries..

Conditions	[formate] / µM
DVH-FDH immmobilized on LGD	3.3 ± 0.4*
DVH-FDH immmobilized on LGD + glutaraldehyde 30 min	4.1
DVH-FDH immmobilized on LGD + glutaraldehyde 60 min	1.5

*average value obtained from 3 experiments.

Table S2. Comparative values of electrocatalytic CO_2 reduction by FDHs immobilized on electrodes.

Enzyme[a]	Electrode	$j_{cat,\ DET\ -0.6\ V}$ (µA/cm²)[b]	$j_{cat,\ MET\ -0.6\ V}$ (µA/cm²)[b]	Γ (mol/cm²)[c]	$k_{cat\ app}$ DET (s⁻¹)	$k_{cat\ app}$ MET (s⁻¹)	Faradaic yield (%)	Operational stability[d]	Reference
W-FDH (*Sf*), ads	$HOPG_{edge}$	-65	-	3.7×10^{-12}	91	-	98	50 % after 3.5 min	23
Mo-FDH (*Ec*), ads	graphite epoxy	-375	-	?	?	-	101.7	not determined	29
W-FDH (*Dv*H), ads	meso TiO_2	-100	-	1.7×10^{-10}	3.0	-	92	92% after 2 h	14
W-FDH (*Me*), crosl	gas diffusion type	-	18,000	$4{,}4 \times 10^{-9}$	-	21	not determined	65% after 5 h	33
W-FDH (*Me*), ads	KB-GCE	-200	-	$8{,}0 \times 10^{-9}$	0.13	-	not determined	not determined	31
Mo-FDH (*Ec*), ent	GCE + redox polymer	-	-62	$1{,}4 \times 10^{-9}$	-	0.22	99	65% after 12 h	35
W-FDH (*Cl*), crossl	C cloth + polyaniline	-2,000	-	$1{,}5 \times 10^{-9}$	7.1	-	92.7	22% after 6 h	34
W-FDH (*Dv*H), ent	gas diffusion + redox polymer	-333	-533	$1{,}0 \times 10^{-9}$	1.7	2.8	inconclusive	80% after 45 h	15
W-FDH (*Dv*H), cov	Functionalized graphite	-160	-375	$8{,}6 \times 10^{-12}$	3.5	8.6	100	34% after 1.5 h	this work

[a]*Sf*: *Syntrophobacter fumaroxidans*. *Ec*: *Escherichia coli*. *Dv*H: *Desulfovibrio vulgaris* Hildenborough. *Me*: *Methylobacterium extorquens*. *Cl*: *Clostridium ljungdahlii*.
[b]current densitive relative to geometric area
[c]Enzyme coverage relative to geometric area measured by QCM or estimated from the total amount of enzyme deposited on the electrode.
[d]Percentage of initial electrocatalytic current retained after a period of time in continuous operation.

References

(1) Brooksby, P. A.; Downard, A. J. Electrochemical and Atomic Force Microscopy Study of Carbon Surface Modification via Diazonium Reduction in Aqueous and Acetonitriile Solutions. *Langmuir* **2004**, *20*, 5038-5045.
